# Controlling the Thermal Stability of Kyanite-Based Refractory Geopolymers

**DOI:** 10.3390/ma14112903

**Published:** 2021-05-28

**Authors:** Juvenal Giogetti Nemaleu Deutou, Rodrigue Cyriaque Kaze, Elie Kamseu, Vincenzo M. Sglavo

**Affiliations:** 1 Local Material Promotion Authority (MIPROMALO), P.O. Box 2396 Yaoundé, Cameroon; kazerodrigue@gmail.com (R.C.K.); kamseuelie2001@yahoo.fr (E.K.); 2 Department of Industrial Engineering, University of Trento, Via Sommarive 9, 38123 Trento, Italy; 3 Laboratory of Applied Inorganic Chemistry, Faculty of Science, University of Yaoundé I, P.O. Box 812 Yaoundé, Cameroon; 4 Dipartimento di Ingegneria dei Materiali e dell’Ambiente, Università di Modena e Reggio Emilia, Via Vignolese 905, 41100 Modena, Italy

**Keywords:** cold setting, kyanite, thermo-mechanical properties, particle size distribution, microstructure

## Abstract

The present project investigated the thermal stability of cold-setting refractory composites under high-temperature cycles. The proposed route dealt with the feasibility of using fillers with different particle sizes and studying their influence on the thermo-mechanical properties of refractory geopolymer composites. The volumetric shrinkage was studied with respect to particle sizes of fillers (80, 200 and 500 µm), treatment temperature (1050–1250 °C) and amount of fillers (70–85 wt.%). The results, combined with thermal analysis, indicated the efficiency of refractory-based kyanite aggregates for enhancing thermo-mechanical properties. At low temperatures, larger amounts of kyanite aggregates promoted mechanical strength development. Flexural strengths of 45, 42 and 40 MPa were obtained for geopolymer samples, respectively, at 1200 °C, made with filler particles sieved at 80, 200 and 500 µm. In addition, a sintering temperature equal to 1200 °C appeared beneficial for the promotion of densification as well as bonding between kyanite aggregates and the matrix, contributing to the reinforcement of the refractory geopolymer composites without any sign of vitrification. From the obtained properties of thermal stability, good densification and high strength, kyanite aggregates are efficient and promising candidates for the production of environmentally friendly, castable refractory composites.

## 1. Introduction

Rapid global population growth and industrialization often conflict with sustainable development and pollution, and environmental drawbacks are typically associated with industrial processes. This particularly applies to the refractory sector, characterized by worldwide production of around 35–40 million tons per year [1,2,3], with applications in industrial sectors including metallurgy, cement kilns, energy treatment and recovery and energy production [4,5,6,7]. The quest to satisfy industrial needs leads to high volumes of refractory production, which is still in conflict with the need for sustainable development in terms of energy demands and carbon footprints. According to the International Energy Agency (IEA) 2008 report and the Global Energy and Climate Outlook 2018 (Report 2018 Global Status), in 2015, the global industrial sector (e.g., Magnesia Spinel Brick Production for production of magnesia refractory raw materials) accounted for 39% of worldwide energy consumption and 21% of total greenhouse gas emissions. To reach the Paris Agreement commitment, a total of 9 Gt per annum CO_2_ reduction is required from the global building sector alone [3,4].

Common refractory materials are processed at high temperatures (≥1400 °C) for prolonged times, leading to high energy consumption and greenhouse gas emissions. For example, the manufacturing of fire clay bricks alone is responsible for the release of 41 tera-grams of CO_2_ into the atmosphere per year [4]. Carbon footprint reduction requirements in refractory processes have addressed research efforts on a global scale to develop novel, ultra-low temperature processing routes. Hereby, the performance (mechanical, thermal stability, crystallography, etc.)/energy cost ratio is a key indicator to successfully reduce environmental impact, thus avoiding the outgrowth of emissions that may arrive as a result of increased material requirements.

Due to their intrinsic chemical and fire resistance, geopolymer binders are considered promising alternatives for producing eco-friendly, non-cementitious composites for civil engineering. Therefore, the development of refractory geopolymers represents important progress [8]. However, their limited stability at high temperatures remains a major drawback. Such restriction is due to the composition of the geopolymer gel or due to the nature of the aluminosilicate used as a solid precursor. Sodium-rich gel favors the crystallization of albite and nepheline, which transforms into a liquid phase at about 900 °C, thus inhibiting its use above 1000 °C [7,8,9,10]. The K-gel is responsible for the presence of crystalline phases like leucite and kalsilite, stable up to 1400 °C, which account for higher mechanical performance. However, larger expansion in the whole system favors the development of cracks in the matrix, thus limiting their use [11]. With respect to such volume instability, which is responsible for cracks and swelling, alternative solutions in the literature involve the use of aggregates as reinforcing elements. These aggregates act as fillers, controlling the thermal stability of the matrix at high temperatures, following the thermochemical transformation phenomena and, consequently, improving mechanical resistance. Among them, the most commonly used are alumina, zirconia, chamotte, corundum, cordierite, porcelain waste, etc. [5,7,12,13,14,15]. Among others, Kohout and Koutnik [16] investigated the effect of four filler types (quartz sand, chamotte, cordierite and corundum) on the thermo-mechanical properties of metakaolin-based geopolymer composites. They revealed that the addition of fillers reduced the thermal shrinkage of the geopolymer products and positively affected the development of mechanical properties. Earlier, in their work focused on the effects of various sizes of tabular alumina particles on the castable microstructure and mechanical properties of geopolymer binders at elevated temperatures, Moosavi and co-workers [9] noticed the crystallization of leucite at 1200 °C and also reported that reduced strength in the same temperature range could be due to thermal expansion mismatch among the liquid phase, leucite and tabular alumina.

Mullite (3Al_2_O_3_·2SiO_2_), cordierite (Mg_2_A_l4_Si_5_O_18_) and kyanite (Al_2_SiO_5_) can represent alternative and inexpensive solutions for reinforcing phases [17,18,19]. Kyanite is a polymorph that transforms at high temperature into mullite, with a small amount of liquid phase between the particles. The transformation of kyanite depends on particle size, impurity content (i.e., iron oxides) and temperature.

The objective of the present study was to investigate the possibility for metakaolin, calcined bauxite, calcined talc and kyanite sieved at 80, 200 and 500 µm to be the raw materials for refractory geopolymers heated at high temperatures (1050, 1150, 1200 and 1250 °C). The scope of the present study is focused on the physicochemical properties, thermal stability and densification parameter behaviors of refractory geopolymers. To achieve this objective, different proportions of kyanite content were applied for each combination of refractory geopolymer mixtures. The flexural strengths were evaluated at different temperature cycles using a material testing system (MTS). The chemical composition and microstructure were characterized by X-ray fluorescence (XRF), scanning electron microscopy (SEM) and X-ray diffraction (XRD). The pore size distribution and phase transformation within the obtained refractory geopolymer products were assessed by means of mercury intrusion porosimeter (MIP) and thermogravimetry analysis (DTA/TG). The proposed route dealt with the feasibility of using fillers with different particle sizes and the study of their influence on the thermo-mechanical properties of the refractory geopolymer composites. Volumetric shrinkage was studied with respect to temperature and particle sizes and amount of fillers.

## 2. Materials and Methods

### 2.1. Materials

Kaolin was collected from Mayououm (West Region, Mayououm, Cameroon) and bauxite from Minim Martarp, Adamawa Region, Martarp, Cameroon. Both raw materials were crushed, milled and sieved below 80 µm and calcined at 700 °C [20,21]. Talc was harvested from Lamal Pougue in the Boumnyébèl area, at about 93 km from Yaoundé (Centre Region, Boumnyébèl, Cameroon). It was crushed and calcined at 700 °C for 4 h; the calcined powder contained 36 wt% MgO, 63 wt% SiO_2_ and 1 wt% Al_2_O_3_. The resulting precursors (metakaolin, calcined bauxite and calcined talc) were used for the preparation of the alkaline binder. Kyanite, to be used as filler, was collected from Dibang, a locality situated in the Centre Region, Cameroon. The kyanite aggregate was oven dried (24 h at 100 °C), ground and crushed to three different particles sizes: 80, 200 and 500 µm. Kyanite with a maximum particle size of 500 µm (coarse aggregates) was obtained after double crushing using a Hammer Mill HM/530/B, Ceramics Instruments, Sassuolo, Italy and sieved at 500 µm. After the first cycle, the retained particles with a size above 500 µm were used for the second cycle. The medium aggregate (200 µm) was prepared with four cycles. After the first cycle, the retained particles were ground up to the fourth cycle. The powder with 80 µm maximum size was obtained through the ball mill process, using a porcelain jar of powder that was crushed in order to have particle sizes below 80 µm. The kyanite materials used in this study have been investigated in previous work, and the chemical composition determined by X-ray fluorescence was 58 wt% Al_2_O_3_, 37 wt% SiO_2_ and 5 wt% Fe_2_O_3_ [20,21].

#### 2.1.1. Preparation of the Alkaline Solution

The alkaline solution used to activate the solid precursors was prepared by mixing potassium hydroxide (KOH) and potassium silicate in specific proportions. 8M KOH (98.66%, Carlo Erba, Milano, Italy) water solution was obtained through dissolution of the hydroxide in distilled water and stirring for 24 h. The potassium silicate was provided by Ingessil, Verona, Italy with a SiO_2_/K_2_O molar ratio of 3.01 and density of 1.38 g/cm^3^. The obtained KOH solution was then mixed with potassium silicate in an equal volume ratio to prepare the final activating solution, according to previous work [8].

#### 2.1.2. Mix-Design of the Refractory Composites

The final matrices for the cold setting refractory were produced with a potassium-based geopolymer gel, and the refractory aggregates, 192 refractory geopolymer samples with variable filler particle sizes (80, 200 and 500 µm) and different amounts of filler ranging from 70 to 85 wt.%, were formulated. Each filler particle possessed 64 samples.

The solid precursor particles (metakaolin–calcined, bauxite–calcined, talc–kyanite) were mixed with the alkaline solution and stirred for 5 min to form the geopolymer mortar. The semi-dry pastes were uniaxially pressed in a steel mold, using a Specac press (London, UK) under 6 tons, to produce disk samples (diameter = 40 mm, thickness = 9 mm). The obtained samples were labelled RB-i, RC-i and RD-i, respectively (i = 70, 75, 80 and 85). Detailed information regarding the formulation of the refractory geopolymer mixtures are reported in Table 1. After pressing, the specimens were immediately sealed into plastic films and cured at room temperature (T = 23 ± 2 °C, relative humidity = 50%) for 7 days. Some specimens were dried at 105 °C and then heated in an electric furnace (Borel FP 1600, Standard Furnaces & Ovens, Grandes-Vies, Porrentruy, Switzerland) at 1050, 1150, 1200 and 1250 °C (heating rate = 5 °C/min, free cooling within the furnace).

### 2.2. Characterization Techniques

#### 2.2.1. Thermal Analysis

Differential thermal analysis and thermogravimetric analysis (DTA/TG) (Model DTA409, NETZSCH, Waldkraiburg, Germany) were used to assess the thermal evolution of the geopolymer samples up to 1600 °C [22,23].

#### 2.2.2. Mineralogical Analysis

Mineralogical analysis was performed using X-ray powder diffractometry (XRD) with a Riguku DMAX III 4057A2 diffractometer (Tokyo, Japan), working at 40 KV and 30 mA of radiation (CuKα, wavelength was 1.5418978 Å). The data were recorded in the 2Ɵ range of 10–70° with a step of 0.03° and dwell time of 10 s. The crystalline phases contained in the refractory geopolymer products were identified by comparison with the PDF (powder diffraction files) standards from ICDD (International Center for Diffraction Data).

#### 2.2.3. Physical and Mechanical Properties

Bulk density and apparent porosity were evaluated according to the ASTM C373-88 norm, using a balance with ±0.0001 g sensitivity. Mean values and standard deviation were calculated using five representative samples. Dimensional stability of the refractory samples was evaluated using a digital Verner caliper. The volumetric shrinkage is given by following Equation (1):

(1) $$V=\frac{V_{f}-V_{i}}{V_{i}}\times 100$$

where *Vi* = average initial volume of four untreated specimens and *Vf* = average final volume of four untreated specimens after exposures at 1050, 1150, 1200 and 1250 °C.

The pore size distribution was measured by mercury intrusion porosimetry (Porosimeter 2000, FISONS Instruments, Glasgow, UK) according to previous work [24,25].

The mechanical performance of the cold setting refractory composite disks was evaluated using the piston-on-three ball method [18]. The tests were carried out at room temperature with a universal mechanical testing machine (MTS810, USA), with load cell of 100 kN and actuator speed of 1mm/min. The flexural strength was determined from the failure load, F, according to:

(2) $$R=\frac{0.3F}{t^{2}}\left[1+2\ln\frac{a}{a}+0.63\left(1-\frac{b^{2}}{2a^{2}}\right)\frac{a^{2}}{R^{2}}\right]$$

where *a* is the radius of the support circle (10.4 mm), *b* the radius of the loading piston (3.35 mm), *R* the radius of the refractory specimens, *t* the thickness of the specimen and *υ* the Poisson’s ratio, assumed equal to 0.23.

#### 2.2.4. Microstructure Analysis

Morphological and structural features of the refractory geopolymer composites were analyzed by scanning electron microscopy (SEM, Joel-JSM5510, Jeol Ltd., Tokyo, Japan), coupled with energy dispersive X-ray spectroscopy (EDS, Joel IT 300), on fragments collected after the mechanical characterization.

## 3. Results and Discussion

### 3.1. Thermal Stability of Refractory Geopolymer Composites under Sintering Cycles

**Figure 1:**
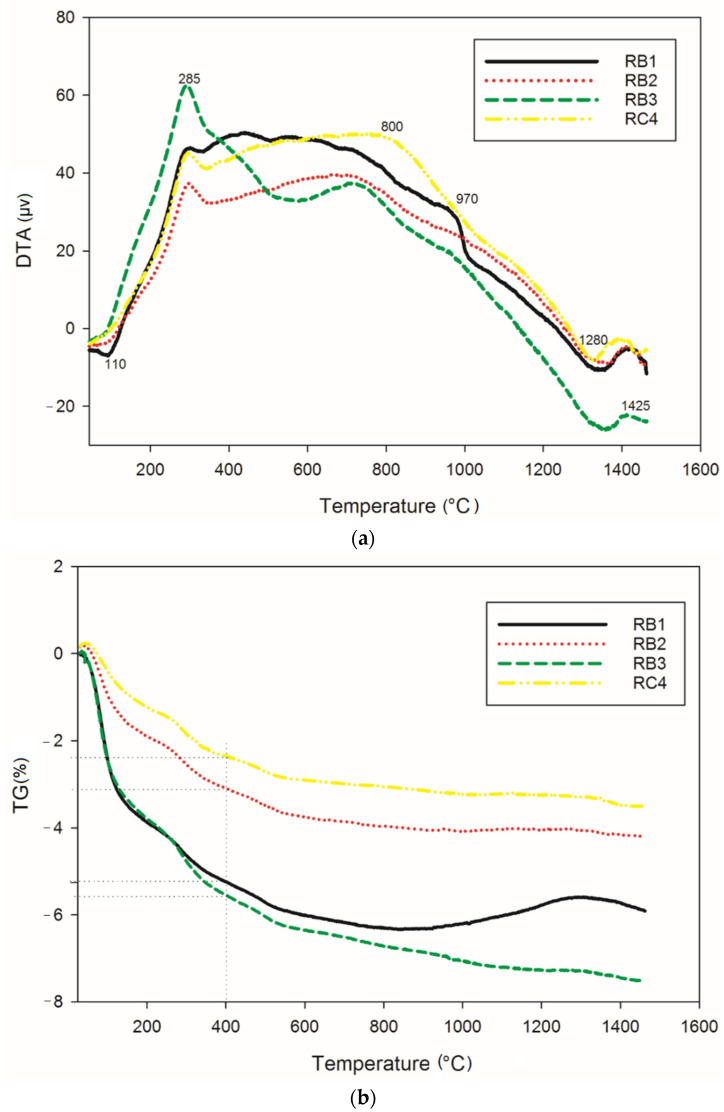
Thermochemical behavior: (**a**) DTA and (**b**) TG.

Figure 1 gathers information on the thermochemical transformations that take place in cold-setting refractory composites RB (containing 70, 75 and 85 wt.% of kyanite, with particle size below 80 µm) and RC1 (containing 70 wt.% of kyanite, with particle size of 200 µm) upon thermal treatment. The thermal behavior of all materials was very similar. The first thermal event (endothermic peak) at 110 °C in the DTA curves was attributed to loss of the absorbed water. The dehydration continued up to about 400 °C, with the corresponding weight loss being 4.9, 2.8 and 2% for RB1 (70 wt.% of kyanite), RB2 (75 wt.% of kyanite) and RB4 (85 wt.% of kyanite), respectively. It was noticed that the amount of fine kyanite particles influenced the mass loss (Figure 1b), with a larger kyanite amount corresponding to better thermal stability. The obtained mass loss was the results of the release of the water out of the geopolymer matrices. In the case of coarser aggregates (RC4), an evident mass loss of 5.3% was observed (Figure 1b). The slight exothermic peak at about 800 °C (RB3) was related to the formation of enstatite and amorphous silicate [26]. Around 970 °C, the presence of mullite crystals, kalsilite, leucite and spinel could be justified by the appearance of an exothermic phenomena (RB1). The decomposition of kyanite particles into mullite and cristobalite started at 1200 °C [17,21]. The nucleation of cordierite crystals from mullite, enstatite and cristobalite was observed with the presence of an endothermic peak at around 1280 °C [6,8,16]. This justified the stability of the refractory matrices (Figure 1b). A further exothermic, solid-state reaction at 1425 °C (Figure 1a) could be likely linked to the recrystallization of the leucite and cordierite. The in situ simultaneous nucleation of the leucite and cordierite justified the transformation of (K–A–S–H) gel to the crystalline K_2_O–MgO–Al_2_O_3_–SiO_2_ above 1300 °C [8,27]. The predominance of crystalline phases, with the formation of mullite, cordierite and kalsilite upon sintering, were significant indicators of the thermal stability of the cold-setting refractory geopolymers.

### 3.2. Dimensional Stability under Thermal Sintering Cycles

**Figure 2:**
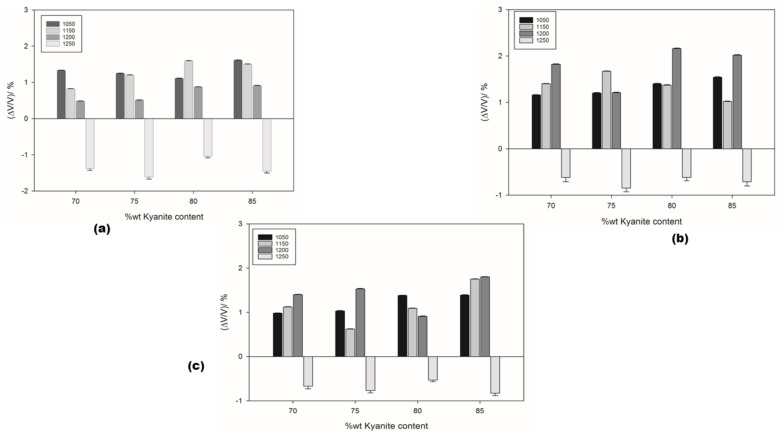
Dimensional stability under sintering cycles: (**a**) RB series; (**b**) RC series and (**c**) RD series.

The thermal shrinkage of the refractory geopolymer composites is shown in Figure 2a–c. At 1050 °C, the shrinkage was 1.33, 1.16 and 0.98 for the RB, RC and RD series, respectively. As the kyanite content increased to 85 wt.%, the shrinkage increased to 1.61, 1.54 and 1.39% for the RB, RC and RD series, respectively. It is worth pointing out that both kyanite particle content and kyanite powder finesses greatly influenced the shrinkage of the resulting matrices. Thus, coarse kyanite aggregates were beneficial for shrinkage at 1050 °C. Larger amounts of kyanite contributed to increasing the shrinkage and the density of the matrices, with the unreacted kyanite particles being important for producing leucite and kalsilite [7,8,28], as pointed out, also, by DTA analysis. Finally, between 1050 and 1250 °C, in all samples, the shrinkage mechanism was mainly governed by the crystallization of mullite and enstatite as well as leucite. Sintering occurred up to 1250 °C; the recorded shrinkage was 1.38, 0.62 and 0.67% for the RB, RC and RD series, respectively, with 70 wt.% of filler (kyanite aggregates). The particle size of kyanite seemed to have less influence on the expansion, decreasing to 1.43, 0.71 and 0.83% with a kyanite load of 85 wt.%. In the present work, the shrinkage of the matrices could be explained by the contact of kyanite particles with the residual viscous phase formed between amorphous silica and alkali metals impurities. The thermal decomposition of kyanite into mullite and silica from 1200 °C induced a shrinkage of 15–20%.

### 3.3. Phase Evolution of the Refractory Geopolymer Composites

**Figure 3:**
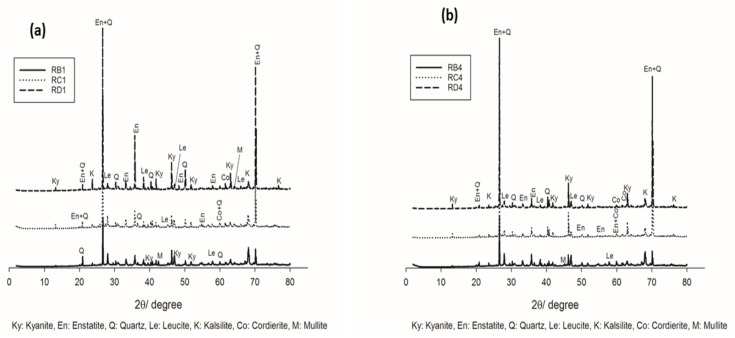
XRD patterns of the refractory geopolymer composites at 1050 °C: (**a**) with 70 wt.%. of kyanite added and (**b**) with 85 wt.% of kyanite added.

**Figure 4:**
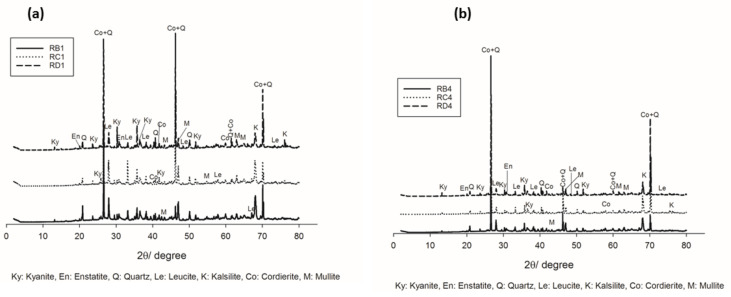
XRD patterns of the refractory geopolymer composites at 1200 °C: (**a**) with 70 wt.% of kyanite added and (**b**) with 85 wt.%. of kyanite added.

Figure 3 and Figure 4 show the XRD patterns of selected refractory geopolymer composites, thermally treated between 1050 °C and 1200 °C. At 1050 °C, the series containing 70 wt.% kyanite aggregates (Figure 3a) contained enstatite (Mg_2_Si_2_O_6_, En, PDF card numbers 86–0435) as the fundamental phase from the thermal transformation of the talc. Leucite (KAlSi_2_O_6_, Le, PDF card numbers 85–1421) and kalsilite (KAlSiO_4_, K, PDF Cards numbers 85-1413) related to the crystallization of the potassium gel were also noticed. Kyanite (Al_2_O_3_.SiO_2_, Ky, PDF cards numbers 11-46) was one of the polymorph minerals, including sillimanite and andalusite, that converted to mullite and cristobalite at relative high temperatures. Accordingly, unreacted particles of kyanite were still present, and their resulting peaks could be easily detected. Traces of quartz (SiO_2_, Q, PDF card numbers 46–1045) and cordierite (Mg_2_A_l4_Si_5_O_18_, Co, PDF card numbers 012–0303) were also observed.

Moreover, for the samples containing 85 wt.% kyanite (Figure 3b), a slight decrease in the intensity of some reflection peaks belonging to the crystalline phases (enstatite, leucite and kalsilite) was observed. This could be attributed to the significant reduction of the liquid phase, which hindered or delayed the nucleation and growth mechanisms and, consequently, limited the crystallization within the matrices.

The increase in the sintering temperature up to 1200 °C favored the crystallization of cordierite (Co) and mullite (M) (Figure 4a,b), in agreement with DTA–TG outcomes. This behavior could be due to the progressive transformation of enstatite and its participation in the formation of cordierite. The presence of mullite could be related to the beginning transformations of the kyanite aggregates within the matrices. Thus, the improvement of crystallization from the progressive formation of the cordierite induced the densification of the specimens with higher mechanical strength. 

### 3.4. Mechanical Properties

**Figure 5:**
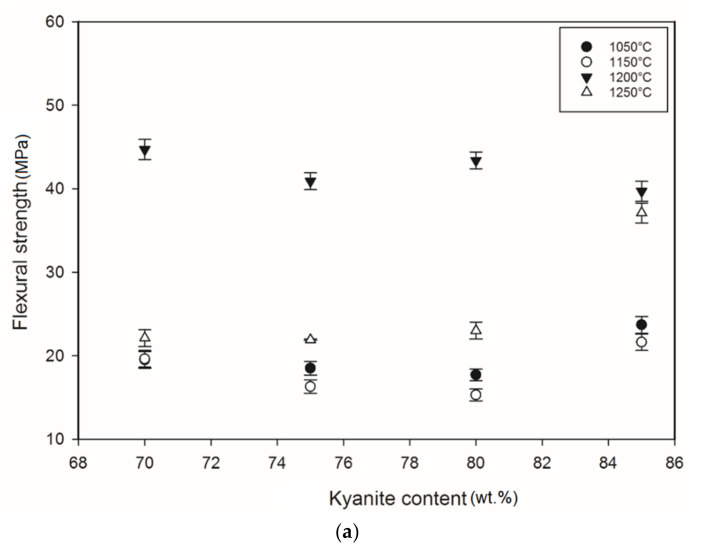

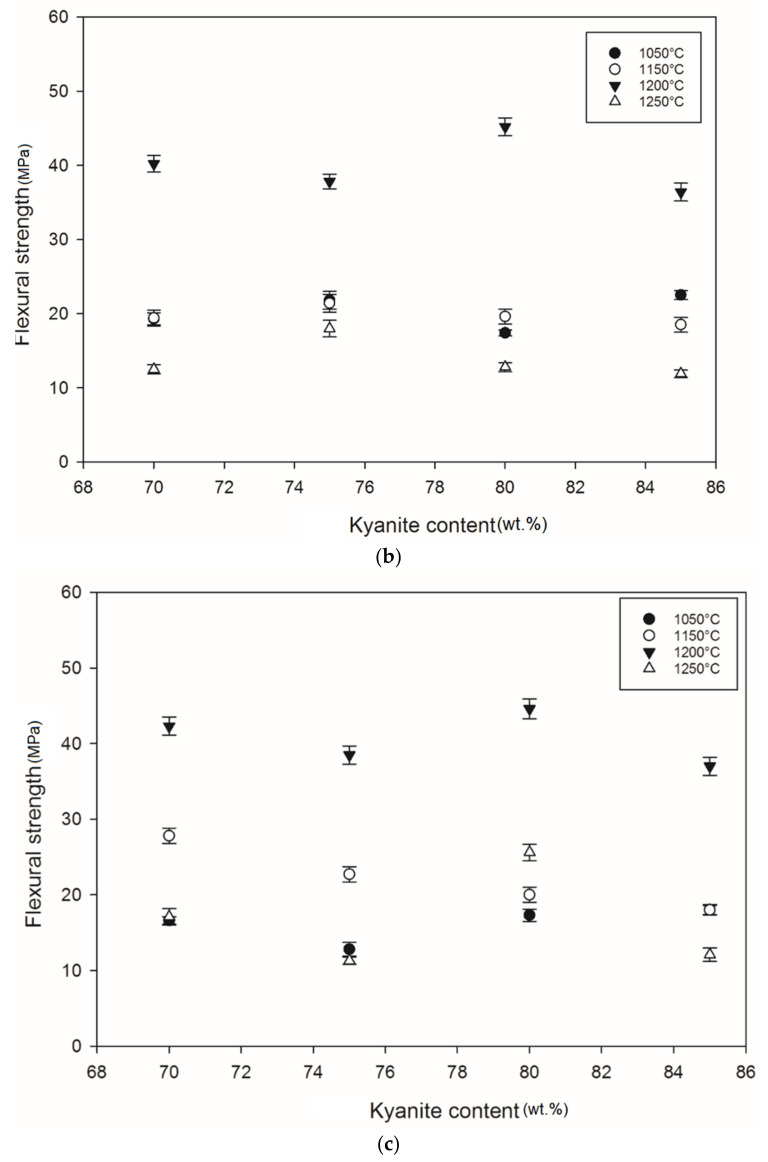
Flexural strength: (**a**) RB series, (**b**) RC series and (**c**) RD series.

Figure 5 shows the flexural strength of the refractory geopolymer specimens after thermal treatment from 105 to 1250 °C. Regardless of the particle size of kyanite aggregates, the flexural strength increased between 1050 and 1200 °C and then decreased. At 1050 °C, in the case of the materials containing 70 wt.% kyanite aggregates, the strengths of RB1 and RC1 were around 20 MPa. These values were slightly high compared to that of the RD1 sample, with a strength of only 16.3 MPa. Larger amount of kyanite aggregates up to 85 wt.% increased the flexural strength to 24, 23 and 18 MPa for RB1, RC1 and RD1, respectively. This meant that high kyanite aggregate content increased the particle density of the fillers in the matrices, which resulted in the improvement of flexural strength. At 1200 °C, with the addition of 70 wt.% kyanite aggregates, the flexural strength significantly increased to 45, 42 and 40 MPa for RB1, RC1 and RD1, respectively. Here, the sintering temperature (1200 °C) appeared beneficial for promotion of the densification process as well as the grain growth and neck bonding among kyanite aggregates, thus contributing to the reinforcement of the refractory geopolymer composite matrices [5,8].

Between 1200 and 1250 °C, the flexural strength dropped down to 22, 13 and 17 MPa for RB1, RC1 and RD1, respectively. These temperatures corresponded to the large volumetric expansion of the refractory specimens (Figure 2). It should be pointed out that the thermal transformation of the kyanite aggregates into mullite and cristobalite seemed to lower the packing density by increasing the inter-particle distance within the matrices [29,30,31]. This phenomenon favored the increase of apparent porosities, which resulted in a decrease in flexural strength to 22, 13 and 17 MPa for RB1, RC1 and RD1, and/or 37, 12 and 12 for 22, 13 and 17 MPa for RB4, RC4 and RD4, respectively.

**Figure 6:**
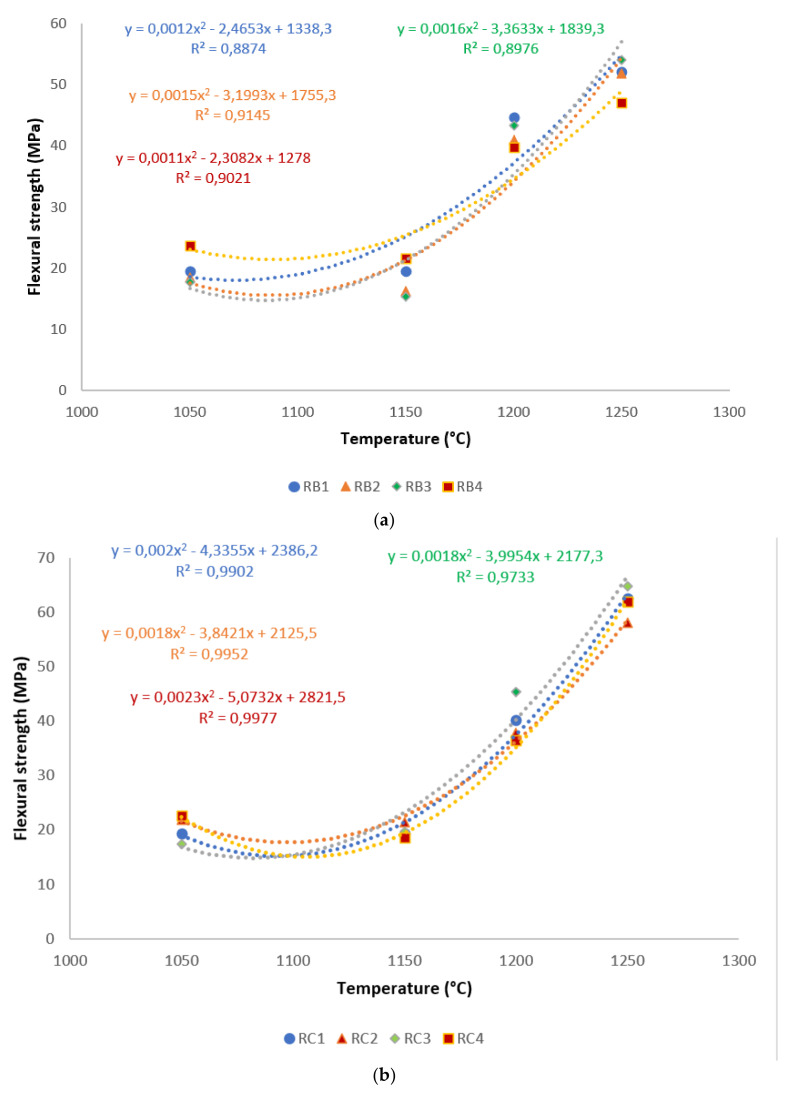

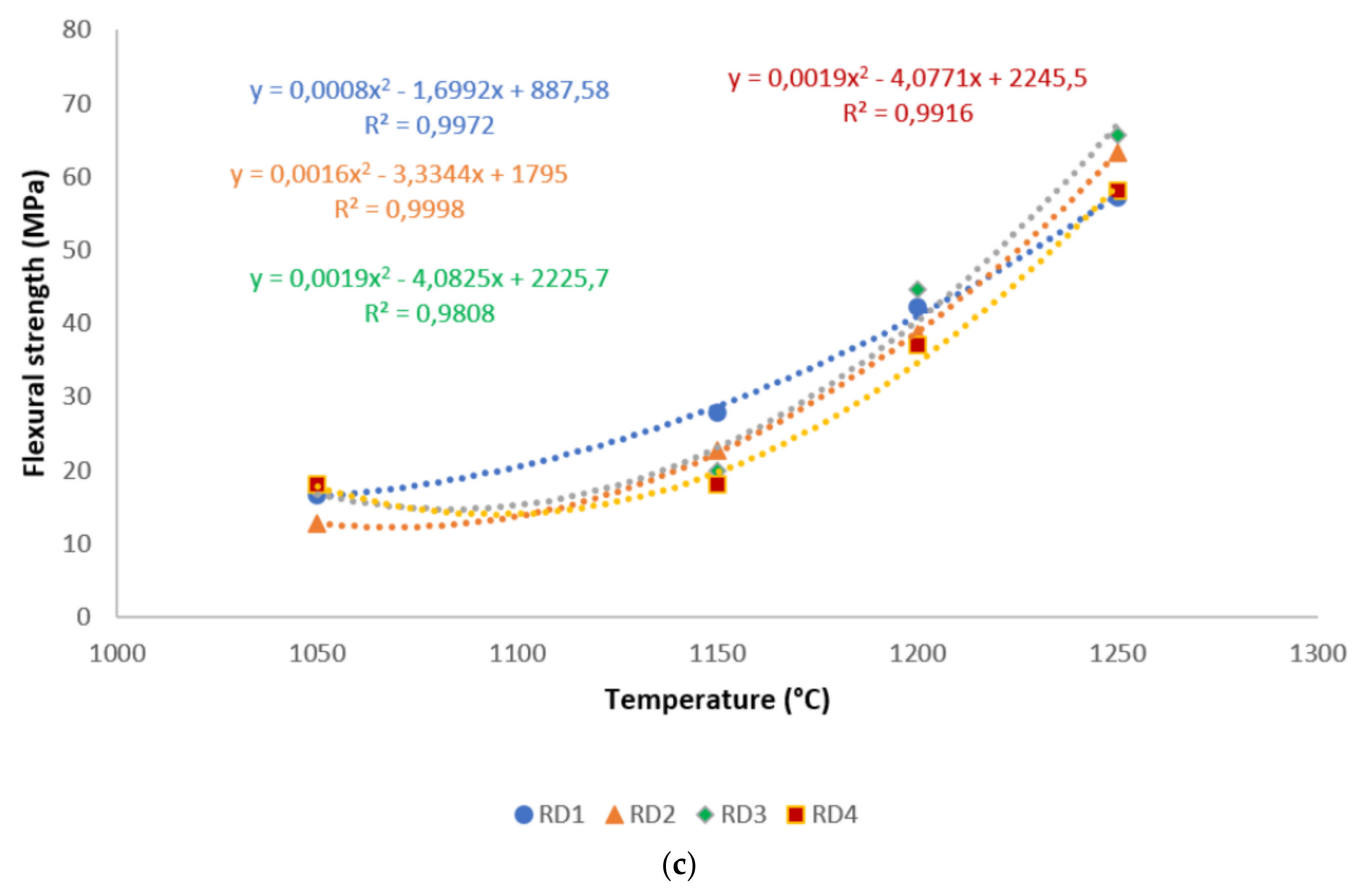
Flexural strength distribution of the refractory geopolymer composites during the sintering cycles: (**a**) RB series, (**b**) RC series and (**c**) RD series.

A correlation is made between mechanical properties, in terms of flexural strength and exposed temperatures, in Figure 6. It is evident from the correlation that there existed a good polynomial relationship between the flexural strength and these sintering cycles. Hence, the equations derived from the correlations could be used in estimating the mechanical properties of refractory geopolymer composites, if one of the properties was known. Excluding RB1 and RB3, for all specimens, the R² was above 0.91, indicating a good agreement between estimated and actual data.

### 3.5. Microstructure

**Figure 7:**
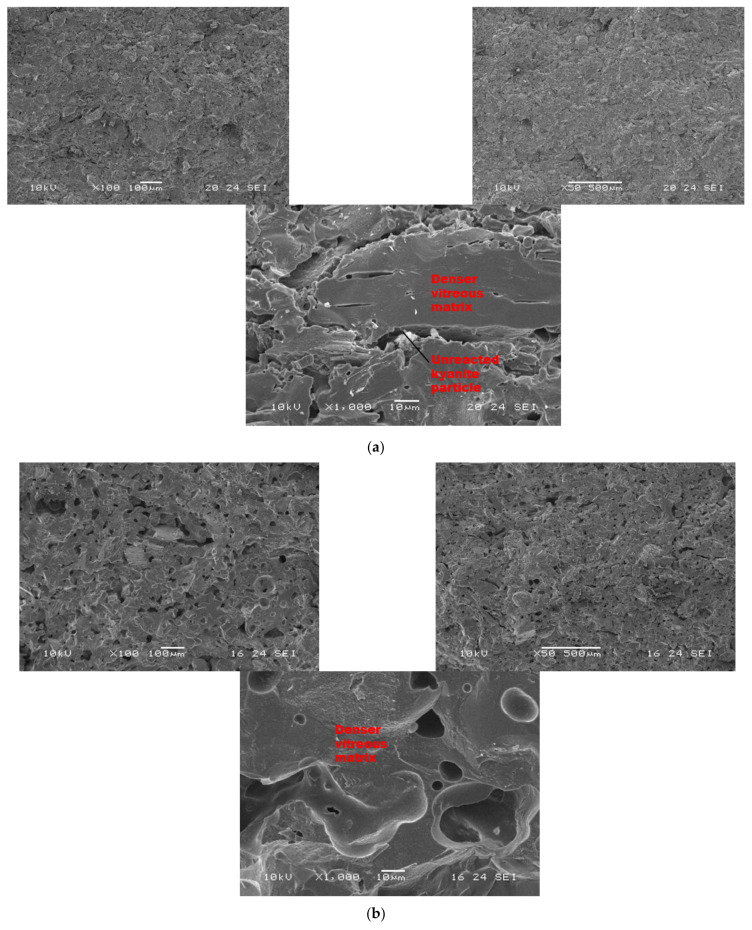

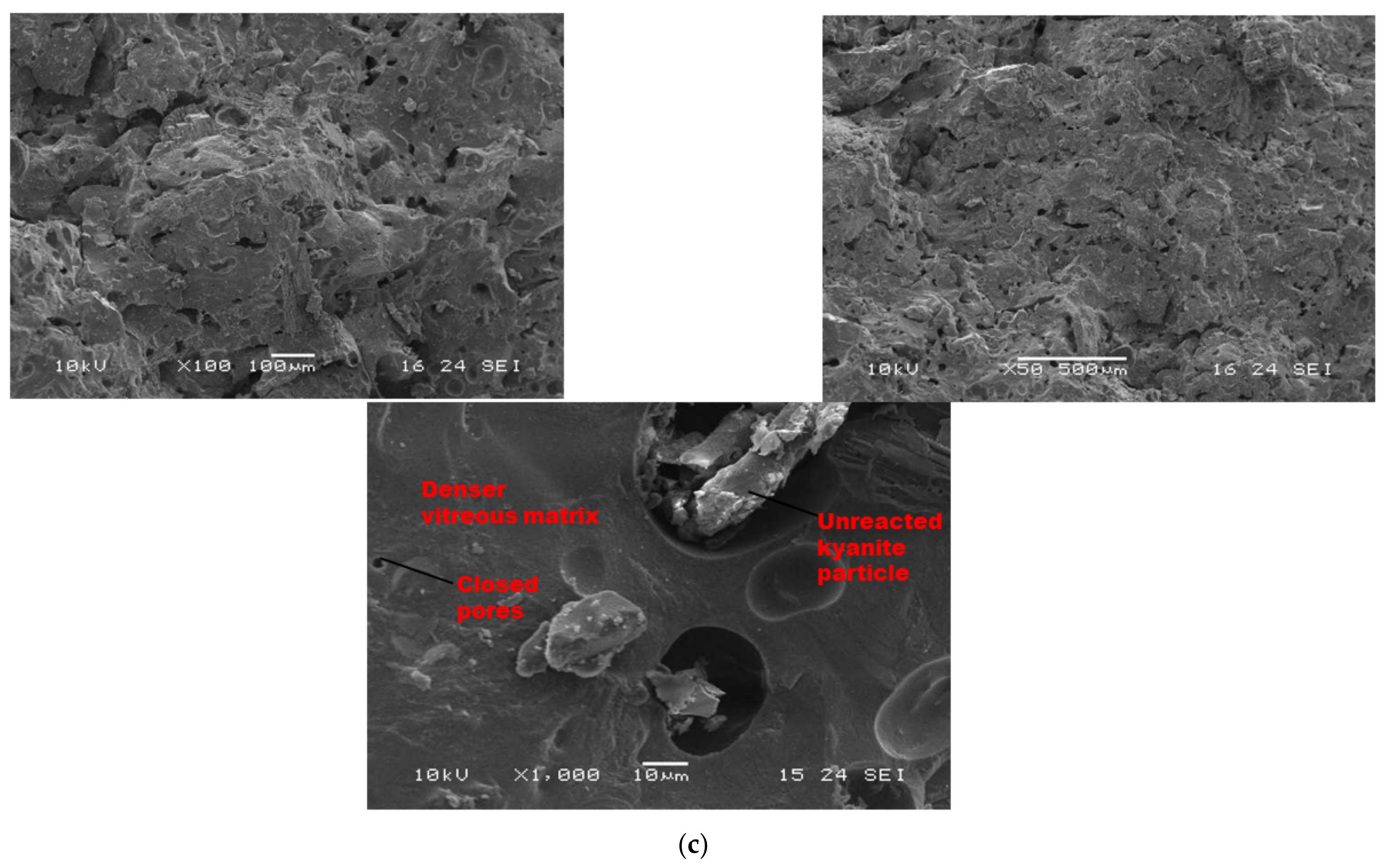
SEM micrographs of selected refractory geopolymer matrices (85 wt.% of kyanite) at 1200 °C: (**a**) RB, (**b**) RC and (**c**) RD.

**Figure 8:**
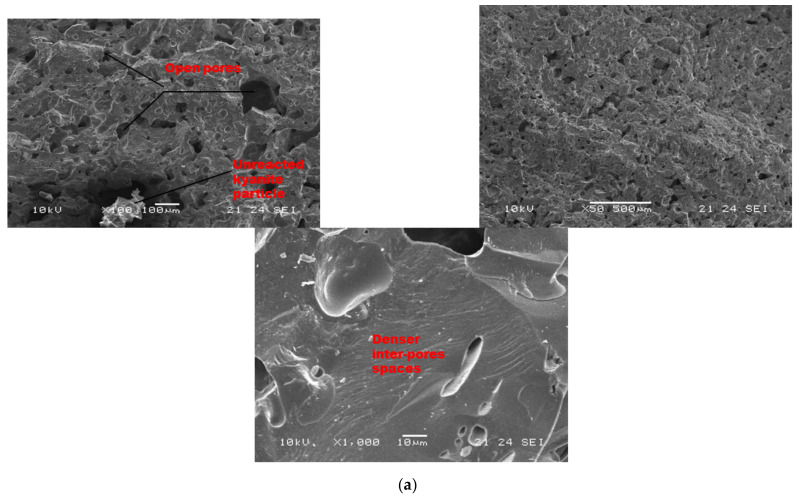

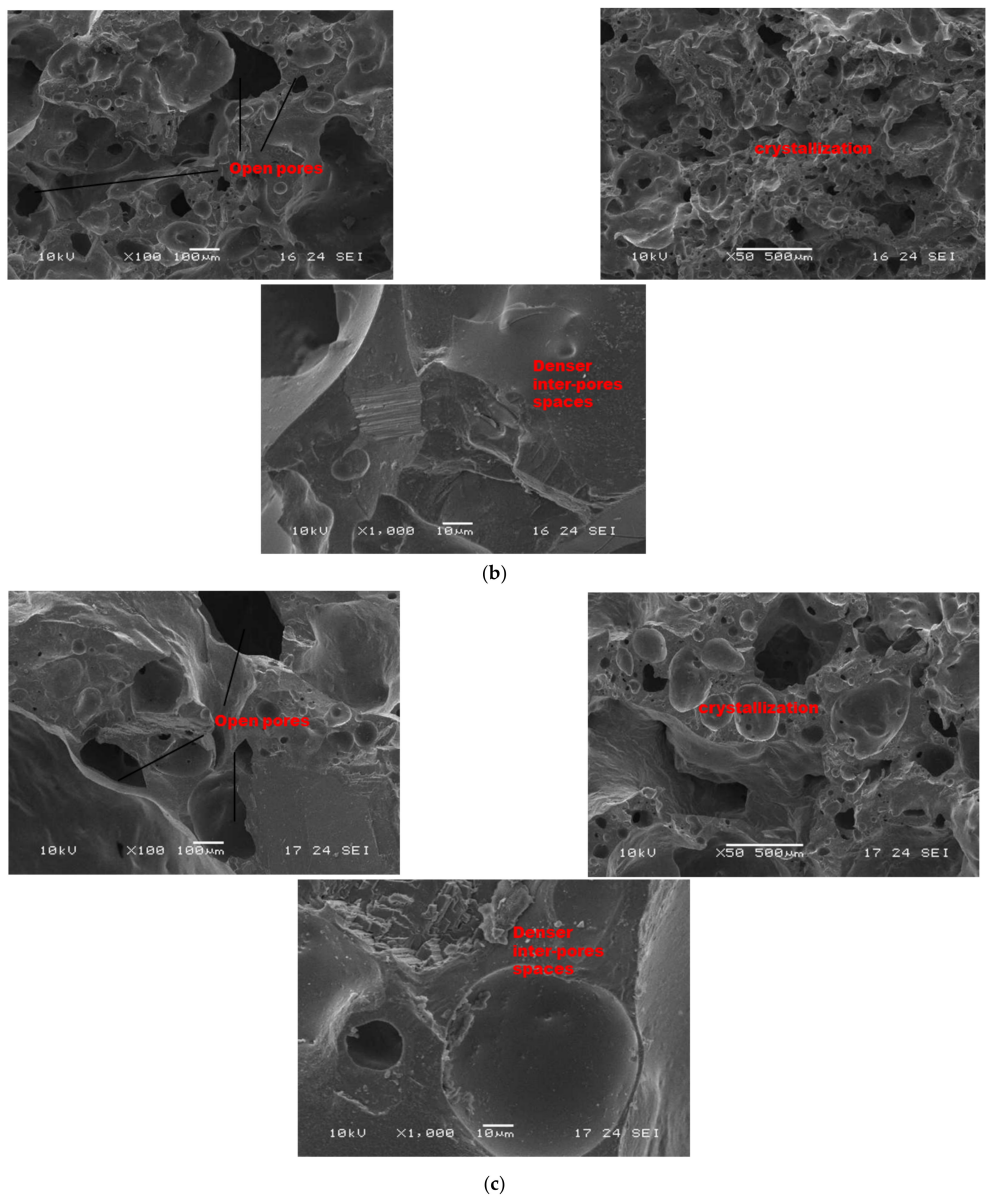
SEM micrographs of selected refractory geopolymer matrices (85 wt.% of kyanite) at 1250 °C: (**a**) RB, (**b**) RC and (**c**) RD.

Figure 7 and Figure 8 show the morphological features of selected refractory geopolymer composites (made with 85 wt.% kyanite) thermally treated at 1200 and 1250 °C. At lower magnification, the refractory geopolymer composites (RB4, RC4 and RD4) heated at 1200 °C exhibited good homogeneous and compact structures (Figure 7a–c). It obviously pointed out the great influence of the finesses of kyanite aggregates on final morphology. Coarser particle sizes of kyanite aggregates lowered the potential of liquid phase formation and favored the pore widening mechanism. The average pore radius was 2, 6.4 and 13.3 µm for the RB4, RC4 and RD4 composites, respectively. This was likely due to the poor neck bonding between the coarse kyanite aggregates and the crystalline phases formed within the matrices. At high magnification (Figure 7), denser and more compact inter-pore spaces are visible. The presence of some unreacted kyanite particles are visible in the matrix. The presence of some closed pores within matrices has been reported in the literature as the volume of the enclosing gases within matter during ceramic processing [32,33,34]. The highly densified inter-pore spaces quite match with the trend of the mechanical properties (Figure 5). 

As sintering temperature increased from 1200 to 1250 °C (Figure 8a–c), porous and homogeneous matrices with a high level of crystallization (cordierite and mullite crystals) appeared. At low magnification (Figure 8), it is noticed that the finesses of the filler slightly affect the level of the crystallization of the matrices. In fact, the thermal transformation of the kyanite aggregates enriched the matrices with mullite and cristobalite. The mechanism determined larger amount of pores in the matrices, thus resulting in the observed high volumetric expansion (Figure 2). The particle size of kyanite aggregates affected the radius of the pores, at 9, 0.3 and 0.3 µm for the RB4, RC4 and RD4 refractory composites, respectively. Higher-magnification micrographs allowed for observing the expansions within the matrices (Figure 8, ×1000).

### 3.6. Pore Size Distribution and Structure

**Figure 9:**
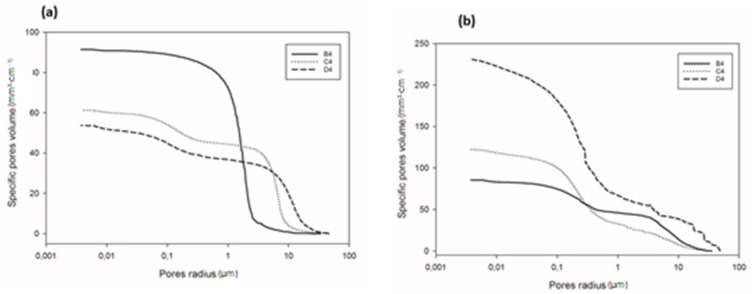
Cumulative pore volume of the cold-setting refractory composites: (**a**) at 1200 °C and (**b**) at 1250 °C.

**Figure 10:**
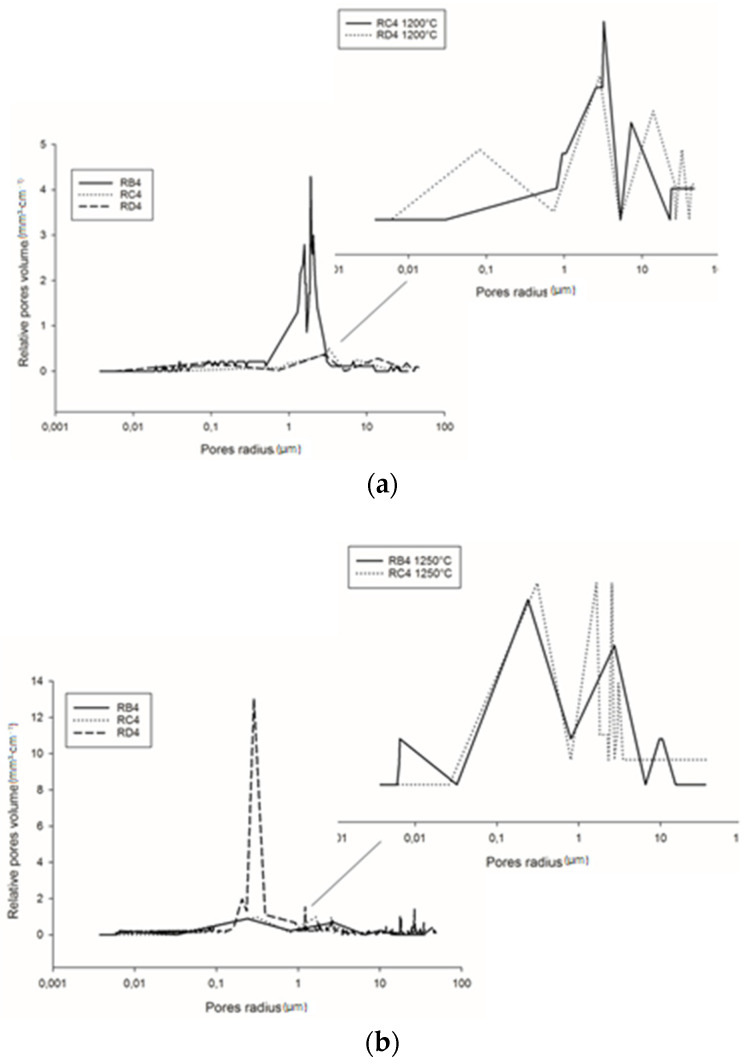
Pore size distribution of the cold-setting refractory composites: (**a**) at 1200 °C and (**b**) at 1250 °C.

Detailed information regarding pore volume and pore size distribution, as well as pore structure in refractory geopolymer composites with 85 wt.% kyanite aggregates, are shown in Figure 9 and Figure 10 and Table 2. From Figure 9a–b, at 1200 °C, the cumulative specific pore volume was 91.4, 61.2 and 53.7 mm^3^·g^−1^ for the RB4, RC4 and RD4 materials, respectively. The specific pore surface area was 0.4, 0.71 and 1.02 m²·g^−1^ for the RB4, RC4 and RD4 specimens, respectively (Table 2). It is worth noticing that the particle size of kyanite aggregates seemed to affect the pores’ characteristics. Coarser kyanite aggregates reduced the cumulative pore volume and favored the development of larger pores with larger surface areas. This was likely due to poor cohesion or voids developed among the coarse grains of kyanite and the formed crystalline phases (mullite, cordierite, leucite, etc.) within the produced matrices [8]. As sintering temperature increased up to 1250 °C, the cumulative pore volume of RB4 specimens decreased to 85.3 mm^3^·g^−1^, while the cumulative pore volume significantly increased to 122.2 and 231.1 mm^3^·g^−1^ for RC4 and RD4. However, the specific pore surface area of RB4 was 1.30 m^2^·g^−1^. Conversely, this parameter increased with the particle size of the filler (Table 2).

Figure 10a shows the pore size distribution of the refractory composites treated at 1200 °C. RB4 specimens show the typical pore size distribution of mullite–cordierite ceramics [34,35,36,37,38,39]. The finesses of the kyanite particles tended to speed up sintering and induce the formation of mullite and cordierite. This induced the formation of pores with bimodal distribution, centered at 1.56 and 2.01 µm. In addition, the use of larger particle sizes of kyanite aggregates changed the pore size distribution. From Figure 9a, it is also observed that the multi-modal distribution for the RC4 and RD4 refractory geopolymer composites shows maxima at about 3.08, 7.7, 23.3 µm and 2.9, 15.8, 34.0 µm for RC4 and RD4, respectively.

At 1250 °C, the thermal decomposition of kyanite aggregates deeply affected pore size distribution (Figure 10b). This corresponded to multimodal distribution in the RB4, RC4 and RD4 matrices. Regardless of aggregate size, the decomposition of the kyanite induced pore coarsening [21,40], with peaks centered at 0.2, 2.7 and 10.5 µm; 0.3, 1.6 and 3.1 µm; and 0.2, 0.3 and 1.2 µm for the RB4, RC4 and RD4 composites, respectively.

**Figure 11:**
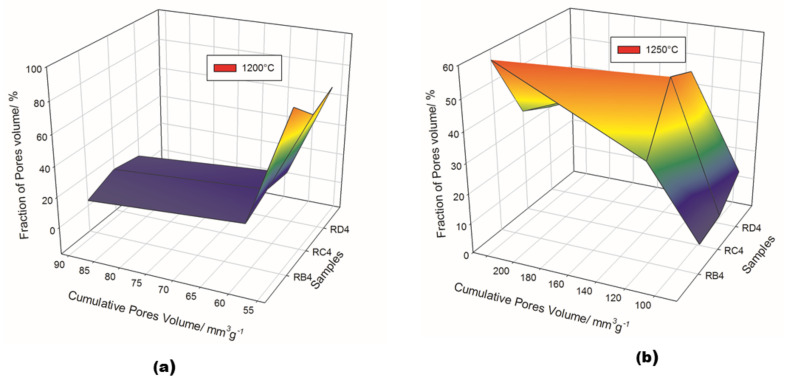
Pore structure of the selected refractory geopolymer matrices. (**a**) 1200 °C, (**b**) 1250 °C.

The pore structure in the synthesized refractory composites is shown in Figure 11 and Table 3. Both the particle size of the fillers and the exposure temperatures seemed to have a great influence on pore volume distribution. Among the specimens treated at 1200 °C, RB4 was characterized by a cumulative pore volume of 91.4 mm^3^·g^−1^, with 3.5% micropores, 10.4% mesopores and 86.1% macropores. The use of coarser kyanite grains caused a slight increase in both micropores and mesopores, the percentages being 11.4, 18.2, 70.4% and 7.9, 15.7, 76.4 % for RC4 and RD4, respectively. This meant that coarser kyanite aggregates were efficient for reducing the amount of voids and cracks within the formulated refractory geopolymer composite matrices.

Despite the relative amount of pore volume at 1250 °C (equal to 85.3, 122.3 and 231.0 mm^3^·g^−1^ for RB4, RC4 and RD4, respectively), the proportion of macropores still decreased to 56.7, 34.8 and 30.5 mm^3^·g^−1^ for the RB4, RC4 and RD4 materials, respectively. This resulted in a remarkable increase in the percentage of mesopores within the matrices, equal to 33.3, 53.5 and 50% for the RB4, RC4 and RD4 samples, respectively. The substantial differences in the pore structures in the whole system between 1200 and 1250 °C were likely correlated with the different particle sizes and the thermal behavior of kyanite filler.

By comparing the achieved results in terms of the types of pores in the present investigation, Table 4 summarizes recent papers focusing on the variation in the fraction of pore volume with the sintering temperature processes of inorganic polymer materials.

### 3.7. Densification and Porosity Evolution

**Figure 12:**
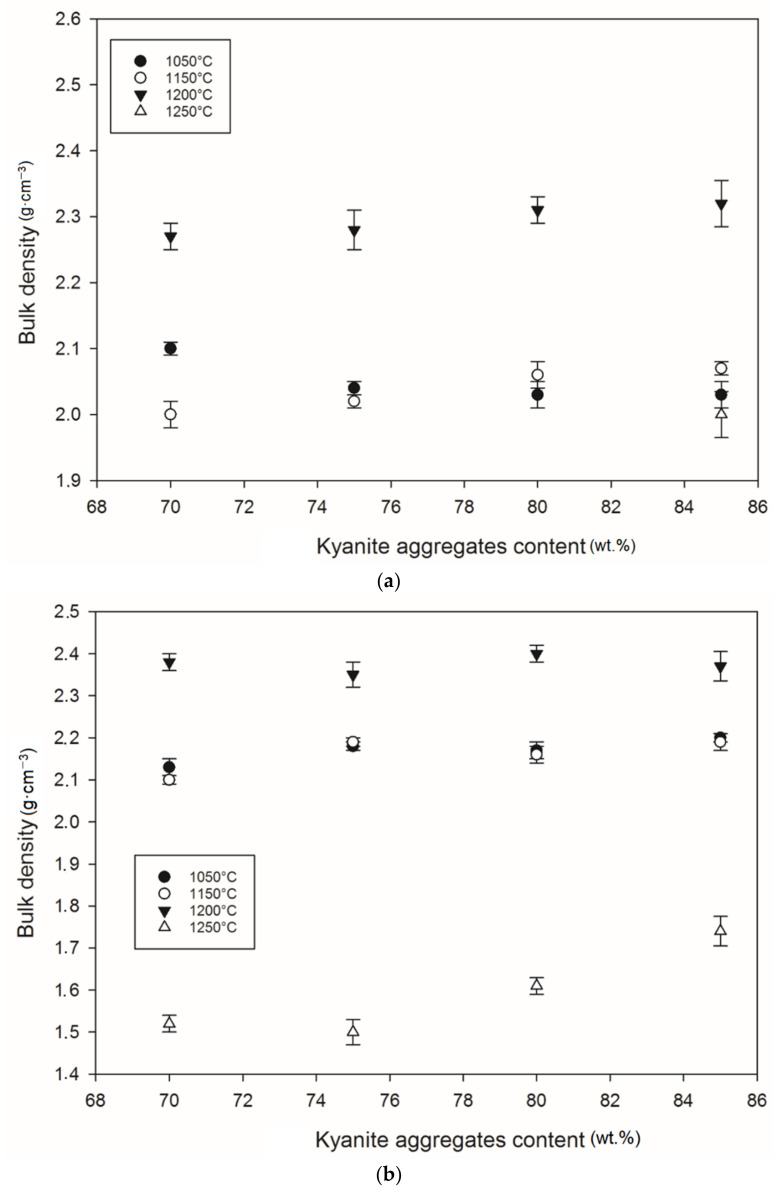

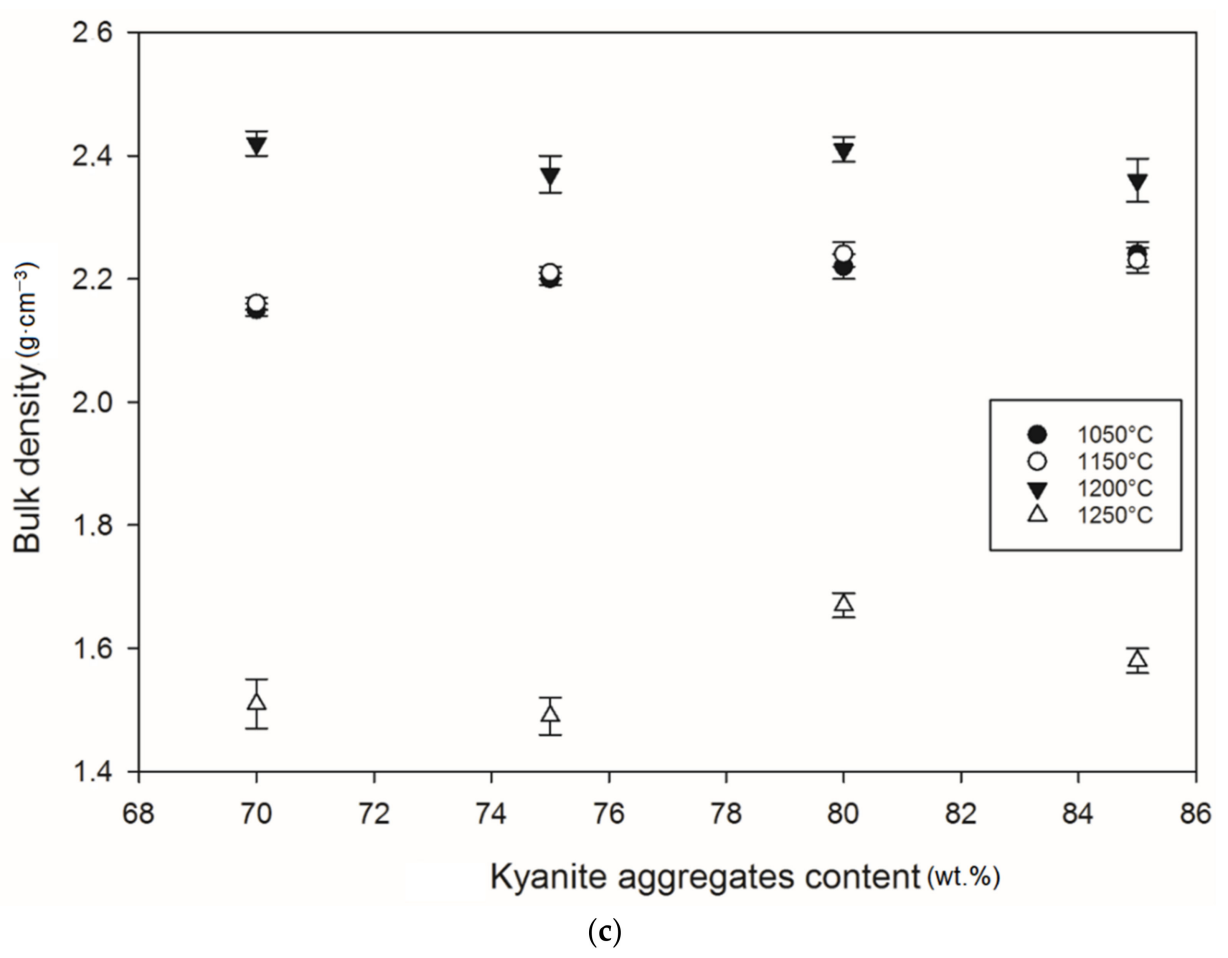
Bulk density behavior: (**a**) RB series, (**b**) RC series and (**c**) RD series.

**Figure 13:**
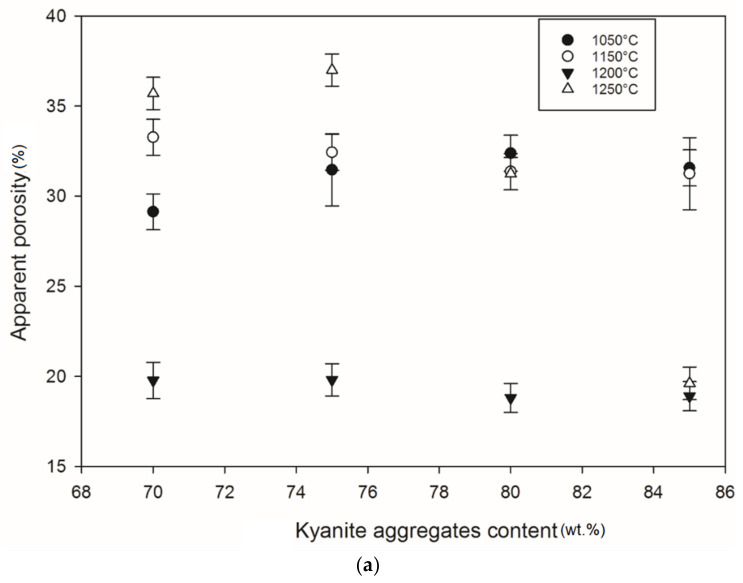

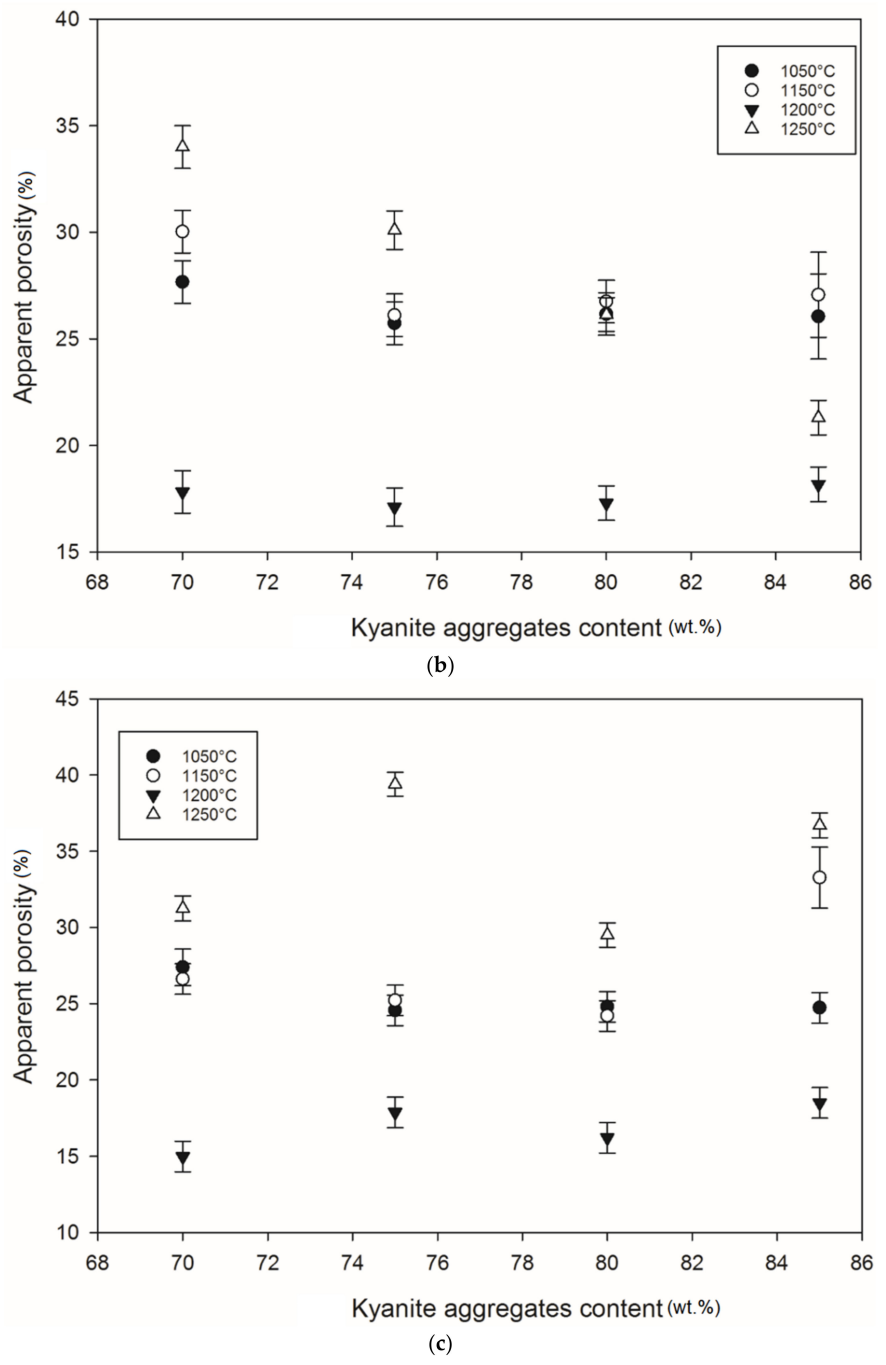
Apparent porosity trend: (**a**) RB series, (**b**) RC series and (**c**) RD series.

Figure 12 and Figure 13 show density and porosity evolution in the cold-setting refractory composites. The effects of kyanite aggregates on densification were not clear. Regardless of the finesses of the filler (kyanite), the bulk density ranged from 1.51 to 2.48 g·cm^−3^. After thermal treatment from 1050 to 1200 °C, a gradual densification appeared because of the crystallization of the potassium geopolymer gel and the formation of new crystalline phases. High bulk density was obtained in the specimens heated at 1200 °C, around 2.27–2.31, 2.35–2.40 and 2.36–2.42 g·cm^−3^ for the RB, RC and RD series, respectively (Figure 12a–c). This tendency could be related to the better connectivity and bonds between the newly formed crystalline phases and the grains of the unreacted kyanite aggregates within the matrices. Such bonding yielded densification, with a good ability to fill pores and voids (Figure 12 andFigure 13). This meant that denser and compact matrices were formed, in agreement with mechanical strength development (Figure 5a–c).

At 1250 °C, the release of additional mullite and cristobalite from the decomposition of kyanite aggregates seemed to create a volumetric expansion and developed conditions for limited contact among the grains, thereby also favoring an increase in the porosity and voids within the matrices. This made the resulting matrices less dense, with high levels of porosity (Figure 12a–c). The apparent porosity ranged from 36 to 19, 34 to 21 and 31 to 37 vol% for the RB, RC and RD series, respectively.

Conclusively, the high achieved porosities combined with their corresponding strength at 1250 °C demonstrates that the synthesized matrices could be indicated for filtering applications where high temperatures are required. However, further analysis should be carried out to assess this hypothesis.

## 4. Conclusions

This study was carried out to explore the feasibility of the use of various sizes of kyanite aggregates as filler in the production of refractory geopolymer composites. The effects of filler content on the formulation, annealing temperature and other thermo-mechanical properties, as well as pore characteristics, were investigated in detail. The outcomes can be summarized as follows:

- Both kyanite content and kyanite powder finesse greatly influenced the thermomechanical performances of the geopolymer matrices subjected to thermal treatments up to 1250 °C;
- The kyanite aggregates were efficient for controlling the melting of the gel binders within the formed matrices;
- At temperatures below 1200 °C, larger amounts of kyanite induced higher particle density within the matrices, resulting in the improvement of flexural strength;
- Coarser kyanite aggregates favored the reduction of pore volume but induced the development of larger pores. This was related to poor cohesion or open voids among the coarse grains of kyanite and the crystalline phases (mullite, cordierite, leucite, etc.) within the matrices.

Conclusively, the above-mentioned findings gave way to predicting the thermal stability of fabricated, refractory, alkali-activated matrices during operating use within a high-temperature environment. Kyanite, as a natural and available material, appears to be an interesting option for the design of cold-setting composites. These matrices are viewed as alternatives for the silico-aluminate refractory composites generally achieved through ceramic processes.

## Figures and Tables

**Table 1:** Formulation of refractory geopolymer mixtures.

Series	Samples	Metakaokin (MK)	Calcined Bauxite (MB)	Calcined Talc (MT)	Kyanite Aggregates	Alkaline Solution/Solid Ratio
**80 µm**	RB1	46.4	12.8	40.52	69.8	0.2
	RB2	46.4	12.8	40.52	74.79	
	RB3	46.4	12.8	40.52	79.76	
	RB4	46.4	12.8	40.52	84.74	
**200 µm**	RC1	46.4	12.8	40.52	69.8	0.2
	RC2	46.4	12.8	40.52	74.79	
	RC3	46.4	12.8	40.52	79.76	
	RC4	46.4	12.8	40.52	84.74	
**500 µm**	RD1	46.4	12.8	40.52	69.8	0.2
	RD2	46.4	12.8	40.52	74.79	
	RD3	46.4	12.8	40.52	79.76	
	RD4	46.4	12.8	40.52	84.74	

**Table 2:** Average pore radius and pore surface area of the refractory geopolymer composites with 85 wt.% kyanite aggregates at 1200 and 1250 °C.

Temperature (°C)	RB4			RC4			RD4		
	APR (µm)	CPV (mm^3^·g^−1^)	PSA (m^2^·g^−1^)	APR (µm)	CPV (mm^3^·g^−1^)	PSA (m^2^·g^−1^)	APR (µm)	CPV (mm^3^·g^−1^)	PSA (m^2^·g^−1^)
1200	2.00	91.4	0.4	6.42	61.2	0.71	13.4	53.7	1.02
1250	9.00	85.3	1.30	0.31	122.3	2.92	0.32	231.0	6.92

**Table 3:** Types of pores of selected refractory geopolymer composites after exposures at 1200 and 1250 °C.

Samples with 85 wt.% of Kyanite Aggregates	Temperature	Pore Size Proportions (%)		
		Micropores (Radius d < 0.1 μm)	Mesopores (1 < Radius < 0.1 μm)	Macropores (Radius ≥ 1 µm)
RB4	1200 °C	3.5	10.4	86.1
	1250 °C	10	33.3	56.7
RC4	1200 °C	11.4	18.2	70.4
	1250 °C	11.7	53.5	34.8
RD4	1200 °C	7.9	15.7	76.4
	1250 °C	19.5	34.8	30.5

**Table 4:** Type of pores of different geopolymers after exposure to high-temperatures (literature results).

Raw Materials/Filler	Exposure Temperatures	Class of Pores	References
>MK, calcined bauxite, calcined talc and kyanite (63, 200 and 500 µm)	1050, 1150, 1200 and 1250 °C	micropores, mesopores and macropores	In this study
Chamotte, alumina–zirconia–silica	800, 1000, 1200 and 1400 °C	micropores, mesopores and macropores	Coppola et al. (2020) [5]
Granulated blast furnace (GBF)	200, 400, 600, 800, 1000 and 1200 °C	gel pores, capillary pores and large capillary pores	Rovnanik et al. (2013) [41]
FA, ground granulated blast furnace slag and sand	200, 400, 600, 800 and 1000 °C	gel pores, capillary pores and large capillary pores	Dudek and Sitarz (2020) [42]
MK, FA and chamotte	200, 400, 600, 800, 1000 and 1200 °C	gel pores, capillary pores and large capillary pores	Rovranik and Safarkova (2016) [43]
Class F fly ash and slag	400, 600 and 800 °C	gel pores and capillary pores	Shaikh (2018) [44]
MK, quartz sand, corundum, chamotte and cordierite	200, 400, 600, 800, 1000 and 1200 °C	gel pores and capillary pores	Kohout and Koutnik (2020) [16]

## Data Availability

The data presented in this study are available on request from the corresponding author.
